# Genetic clusters and sex-biased gene flow in a unicolonial *Formica *ant

**DOI:** 10.1186/1471-2148-9-69

**Published:** 2009-03-31

**Authors:** Barbara Holzer, Laurent Keller, Michel Chapuisat

**Affiliations:** 1 Department of Ecology and Evolution, University of Lausanne, Biophore, UNIL-Sorge, 1015 Lausanne, Switzerland

## Abstract

**Background:**

Animal societies are diverse, ranging from small family-based groups to extraordinarily large social networks in which many unrelated individuals interact. At the extreme of this continuum, some ant species form unicolonial populations in which workers and queens can move among multiple interconnected nests without eliciting aggression. Although unicoloniality has been mostly studied in invasive ants, it also occurs in some native non-invasive species. Unicoloniality is commonly associated with very high queen number, which may result in levels of relatedness among nestmates being so low as to raise the question of the maintenance of altruism by kin selection in such systems. However, the actual relatedness among cooperating individuals critically depends on effective dispersal and the ensuing pattern of genetic structuring. In order to better understand the evolution of unicoloniality in native non-invasive ants, we investigated the fine-scale population genetic structure and gene flow in three unicolonial populations of the wood ant *F. paralugubris*.

**Results:**

The analysis of geo-referenced microsatellite genotypes and mitochondrial haplotypes revealed the presence of cryptic clusters of genetically-differentiated nests in the three populations of *F. paralugubris*. Because of this spatial genetic heterogeneity, members of the same clusters were moderately but significantly related. The comparison of nuclear (microsatellite) and mitochondrial differentiation indicated that effective gene flow was male-biased in all populations.

**Conclusion:**

The three unicolonial populations exhibited male-biased and mostly local gene flow. The high number of queens per nest, exchanges among neighbouring nests and restricted long-distance gene flow resulted in large clusters of genetically similar nests. The positive relatedness among clustermates suggests that kin selection may still contribute to the maintenance of altruism in unicolonial populations if competition occurs among clusters.

## Background

Many social animals live in small family groups of closely related individuals, and the role of kinship in promoting the evolution of reproductive altruism and eusociality by kin selection has long been recognized [1-4]. However, some ant species have an extraordinary social organization, called 'unicoloniality', which is characterised by an absence of behavioural boundary among interconnected nests that contain many queens and exchange workers, brood and fertile queens [5].

The evolution and maintenance of unicolonial populations constitutes one of the enduring major challenges for kin selection, and more generally for modern, gene-centred evolutionary theory [6-9]. This is because high queen number and mixing of individuals among nests can lead to extremely low relatedness among nestmates [6,8,10,11]. In short, if workers help completely unrelated individuals, selection should favour selfish females that develop into queens or produce males, and adaptive altruistic worker behaviour cannot be maintained by kin selection [6]. Hence, the long-term maintenance of altruism critically depends on the actual degree of relatedness among nestmates, which in turn depends on the number of breeders, dispersal of queens and males, and scale at which competition takes place [12-14]. It is therefore of considerable interest to study the breeding system, dispersal pattern and genetic structuring in unicolonial populations.

Studies of unicolonial ants have mostly focussed on invasive species such as the argentine ant *Linepithema humile *[9,15-17], the fire ant *Solenopsis invicta *[18], or the little fire ant *Wasmannia auropunctata *[19,20]. In their invasive range, some of these unicolonial species form very large supercolonies [9,15,19]. Supercolonies constitute closed breeding units that are large enough to prevent direct interactions between individuals from distant nests within the same supercolony [9]. Members of supercolonies are aggressive towards members of other supercolonies, but not towards members of their supercolony. In their native range, *W. auropunctata *seems to be multi-colonial [19], whereas *L. humile *forms supercolonies of much smaller size than in the introduced range [9]. If these small supercolonies are closed breeding units that compete which each other, explaining the evolution and stability of unicoloniality becomes less problematic [9].

Several native, non-invasive ant species have been reported to form unicolonial populations in the genera *Formica *[21-25], *Lasius *[7,26], *Myrmica *[27-30], *Tetramorium *[31] and *Polyrhachis *[32]. Interestingly, most of these populations of native species do not seem to have clear-cut behavioural boundaries (i.e. aggression) delineating supercolonies, even over large geographical areas in some cases [e.g. [7,33]]. Moreover, several of the non-invasive unicolonial ant species show significant isolation by distance within populations [i.e. "population viscosity", [1,22,29,32]].

Hamilton [1] first proposed that population viscosity due to limited dispersal of individuals from their birth place increases the relatedness among interacting individuals, and may thus favour the evolution of altruistic behaviour. However, formal models show that this effect critically depends on the definition of altruism and on details of the life cycle, such as if altruism is provided by workers or focal queens, and occurs before or after dispersal [34-36]. Altruism is more likely to evolve when workers provide it, when there are few breeders, and when the beneficiaries of altruism disperse (e.g. winged queens and males). When altruists directly compete with the individuals they help, the benefits of helping related individuals is reduced by increased competition among relatives [13,36,37]. However, if the number of queens is moderate and migration restricted, population viscosity can promote the evolution of altruistic sterile workers under a simple life-cycle in which workers help queens after dispersal [34].

These models show that the impact of population genetic structure on the evolution of altruism critically depends on specificities of the life-cycle, and in particular on the patterns of dispersal and behavioural interactions of queens, males and workers. It is therefore important to obtain detailed empirical data on sex-specific dispersal and on cryptic genetic discontinuities that may correspond to cooperative units in unicolonial species. Such data will be useful to develop new models and design behavioural studies that will help to assess the role of kin selection in structured population.

Here, we focus on the genetic structure of *Formica paralugubris*, a wood ant native to the European alpine area. Each nest contains hundreds of reproductive queens [22,38,39]. Winged queens and males are produced once per year in spring, and a mating flight occurs in early summer. *Formica *workers have retained ovaries and are able to lay haploid eggs, but the proportion of egg-laying workers varies across species and worker-laid brood can be policed by other workers [40]. It would be of great interest to study whether *F. paralugubris *workers gain direct fitness by producing males. However, such studies are hampered by the difficulty to detect worker-produced males with genetic markers when there are many queens per nest.

In *F. paralugubris*, young queens have alternative reproductive strategies [41]. They can mate and stay within their natal nest [41,42], or fly to open meadows where they mate before seeking adoption in established nests of *F. paralugubris *or *Serviformica *species [41]. Later on, some adult dealate queens may also disperse on foot to join neighbouring nests or establish new nests with the help of workers [colony budding, [11,22]].

The dispersal behaviour of males has been little studied so far. They may mate within their natal nest or fly away to mate on open meadows, or possibly in other nests [41]. In addition to direct male dispersal, any flight by mated queens will also contribute to increase male gene flow, as sperm is carried along in the spermatheca of queens. A comparison of biparentally-inherited nuclear genetic markers and maternally-inherited mitochondrial markers would permit to evaluate the relative contribution of each sex to gene flow in this ant species with complex and potentially sex-biased dispersal [e.g. [43-45]]. The estimates of numbers of migrants should however be considered with caution and only compared in relative terms, particularly when the markers differ in their levels of genetic variation [46,47] and the system departs from the assumptions of an island model [48].

*F. paralugubris *forms unicolonial populations, as demonstrated by the very high number of queens per nest, movement of queens and workers among neighbouring nests, and lack of aggression between workers from distant nests within populations [22,33,38,39,49]. Microsatellite analyses in one population revealed that distant nests were genetically differentiated, resulting in low but significant relatedness among nestmate workers [22,42]. This genetic differentiation indicates that long-distance gene flow between established nests is limited and that most young queens mate and stay within or close to their natal nest [42]. Importantly, such restricted dispersal of queens and males may produce clusters of related nests.

The development of Bayesian clustering methods for population genetics now enables the detection of cryptic genetic population structure using the genotypes of individuals as the sole source of information [50,51]. In short, these methods attempt to partition individuals into groups at Hardy-Weinberg equilibrium. In a recently developed extension of these methods, geo-referenced individuals are partitioned into sets of panmictic populations, which allows to detect the spatial location of cryptic genetic discontinuities [52]. These new tools are especially useful in species like unicolonial ants where no apparent landscape and behavioural borders are present among nests.

The main aim of our study was to investigate and compare the fine-scale population genetic structure of three unicolonial populations of *F. paralugubris*. We therefore developed mitochondrial markers that, together with the nuclear microsatellite markers available, allowed us to detect cryptic genetic clusters of nests, obtain more precise information on genetic differentiation within populations, and separate male from female gene flow. These new data should help to evaluate the role of kin selection in native unicolonial populations.

## Results

### Haplotype distribution

**Figure 1:**
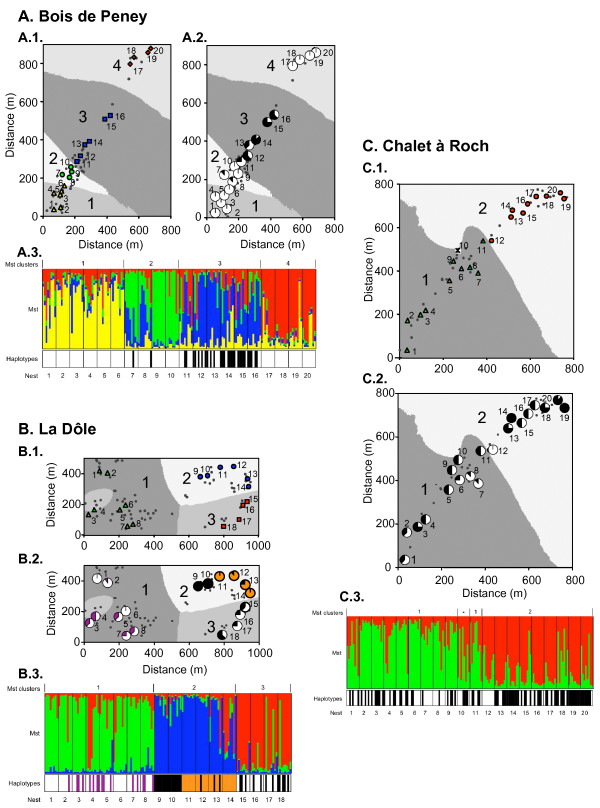
**Genetic clustering of nests**. The cryptic genetic structure of three populations of *F. paralugubris *at Bois de Peney (A), La Dôle (B) and Chalet à Roch (C). Panels 1 (A.1, B.1 and C.1) show synthetic maps of the clusters detected when analysing the microsatellite genotypes with the program Geneland. Large numbers indicate the cluster, grey tones the cluster area, and small numbers the sampled nests. Symbols in color represent sampled nests belonging to the same cluster, and small black dots indicate nests in the vicinity of sampled nests. The asterisk in the population Chalet à Roch (C.1) indicates a nest with a posterior probability close to 0.5 to belong to one or the other cluster. Panels 2 show the distribution and frequency per nest of the mitochondrial haplotypes (violet: H1; orange: H2; white: H3; black: H4). The upper parts of panels 3 (Mst) show the population structure detected when analysing the microsatellite genotypes with the program Structure. Each individual is represented by a vertical line, which is partitioned into *k *coloured segments that represent the individuals's estimated membership fractions in *k *clusters. The bottom parts of panels 3 (Haplotypes) show the haplotype for each individual, given as a violet (H1), orange (H2), white (H3) or black (H4) bar. Thin lines in panels 3 separate individuals from different nests. Microsatellite clusters in panels 2 and 3 correspond to the ones detected with Geneland in panels 1.

We screened approximately 5165 bp of the mitochondrial DNA of *F. paralugubris *for sequence polymorphisms in each of 11 individuals coming from 11 nests spread over seven populations and two mountain ranges. Despite the large distance of up to 76 km among sampled individuals, we detected only three polymorphic sites that, in combination, resulted in four haplotypes (H1 to H4). All four haplotypes were present in the population at La Dôle. Three haplotypes (H1, H3, H4) were found at Champs Simon, and two haplotypes (H3, H4) at Bois de Peney and Chalet à Roch. We found only one haplotype (H3) in the population at Bois de Ban, and another single haplotype (H1) in the two alpine populations (Château d'Oex and Lac de l'Hongrin). We detected two haplotypes in many of the nests (40%, 67% and 85% at Bois de Peney, La Dôle and Chalet à Roch, respectively; Fig. 1 panels 2 and 3), which indicates that nests occasionally accept foreign queens.

### Genetic clustering of nests within populations

The analyses of the microsatellite genotypes with the program Geneland revealed the presence of cryptic genetic clusters of nests (Fig. 1 panels 1). The number of inferred clusters was the same in all ten runs performed for each population, with posterior probabilities ranging from 53 to 72%. The number and composition of clusters was identical when analysing the data with the program Structure, which confirms that the clustering obtained with Geneland was robust and did not depend on the spatial position of nests. The analysis with Structure also showed some admixture among clusters, as several individuals had a large fraction of their genotype assigned to a cluster other than the one in which they were sampled (Fig. 1 panels 3).

The amount of genetic differentiation among nest clusters varied among populations, but was significant in all cases (Table 1). The limits of the nest clusters based on microsatellite genotypes largely coincided with discontinuities in the frequency distribution of the mitochondrial haplotypes (Fig. 1 panels 2 and 3). As a result, each population exhibited significant mitochondrial DNA haplotype frequency differentiation among the microsatellite-based clusters of nests (Table 1).

**Table 1:** Genetic differentiation among nests and among clusters

		*Microsatellites*					*Mitochondrial*	
			
Population	*k*	*F*_ind-nest_	*F*_ind-cluster_	*F*_ind-pop_	*F*_nest-cluster_	*F*_cluster-pop_	*F*_nest-cluster_	*F*_cluster-pop_
Bois de Peney	4	-0.026 [-0.044; -0.006]	0.003 [-0.028;0.031]	**0.069** [0.034; 0.098]	**0.027** [0.008; 0.044]	**0.066** [0.049; 0.081]	**0.089**	**0.450**
La Dôle	3	-0.025 [-0.064; 0.015]	0.022 [-0.027; 0.067]	**0.120** [0.064; 0.179]	**0.044** [0.022; 0.072]	**0.100** [0.076; 0.130]	**0.345**	**0.431**
Chalet à Roch	2	0.005 [-0.028; 0.043]	0.034 [-0.003; 0.079]	**0.071** [0.022; 0.119]	**0.029** [0.015; 0.045]	**0.037** [0.014; 0.062]	**0.165**	**0.083**

*F*-analyses of variance of the differentiation at microsatellite and mitochondrial markers for each population. The clusters were previously defined by analysing the microsatellite genotypes with the program Geneland. *k *is the number of clusters. *F*-estimates significantly greater than zero are in bold and 95% confidence intervals are given in brackets.

The population at Bois de Peney had the highest number of clusters, as four genetically distinct groups of nests emerged when analysing the microsatellite genotypes with Geneland (p = 52.7%, Fig. 1 panel A.1) and Structure (Fig. 1 panel A.3). Three of these nest clusters were almost exclusively composed of individuals with the same mitochondrial haplotype, whereas one cluster had a high frequency of another haplotype (Fig. 1 panels A.2 and A.3). The population at La Dôle had three well-defined nest clusters based on microsatellite differentiation (p = 72.0%, Fig. 1 panels B.1 and B.3). The four mitochondrial haplotypes were unevenly distributed among these clusters: we detected only two haplotypes per cluster, and two haplotypes were restricted to single clusters (Fig. 1 panels B.2 and B.3). Finally, the population at Chalet à Roch had two rather ill-defined nest clusters based on microsatellite differentiation (p = 60.0%, Fig. 1 panels C.1 and C.3), with many of the individuals having a large fraction of their genotype assigned to the other cluster (Fig. 1 panel C.3). Two mitochondrial haplotypes intermingled throughout the transect, but had different frequencies in each cluster (Fig. 1 panels C.2 and C.3).

The hierarchical *F*-analyses of variance of the microsatellite genotypes revealed significant genetic differentiation among nests within clusters and among clusters within populations at our three study sites (Table 1). The lowest genetic differentiation among clusters was found at Chalet à Roch and the highest at La Dôle (*F*_cluster-pop _= 3.7% and 10%, respectively). The significant *F*_ind-pop _coefficient indicates non-random mating at the population level. This deficit of heterozygotes is consistent with a Wahlund effect over small spatial scales due to genetic differentiation among nests and among clusters. In contrast, *F*_ind-cluster _was not significantly different from zero, which is in agreement with the hypothesis of random mating within clusters that underlies the way clusters are identified. This result suggests that clusters roughly correspond to breeding units in Hardy-Weinberg equilibrium, despite the significant genetic differentiation among nests. Finally, slightly negative and close to zero *F*_ind-nest _coefficients are consistent with the expected excess of heterozygous within brood coupled with high number of queens that mate at random within nests.

In line with the significant genetic differentiation among nests and clusters, the relatedness among nestmate workers and the relatedness among clustermates measured with respect to the allele frequencies in the total population were significantly larger than zero in all three populations (Table 2). Interestingly, the relatedness among clustermates was fairly high, and accounted for most of the relatedness among nestmates measured with respect to the total population. Conversely, the relatedness among nestmates measured with respect to the cluster was moderate, but still significantly greater than zero in each population (Table 2). Together, these results reveal a multi-level pattern of genetic structure, with fairly high gene flow among neighbouring nests within clusters and restricted gene flow among clusters within unicolonial populations. This additional level of structure above the nest level results in a high relatedness among members of the same cluster.

**Table 2:** Estimates of genetic relatedness

Population	*n*	*r*_nestmates (cluster)_	*r*_clustermates (population)_	*r*_nestmates (population)_
Bois de Peney	20	**0.054 ± 0.017**	**0.130 ± 0.019**	**0.149 ± 0.019**
La Dôle	18	**0.092 ± 0.035**	**0.185 ± 0.028**	**0.209 ± 0.030**
Chalet à Roch	20	**0.059 ± 0.021**	**0.075 ± 0.022**	**0.093 ± 0.016**

*r*_nestmates (cluster) _is the relatedness among nestmates with respect to allele frequencies in the cluster (± standard error). *r*_nestmates (population) _or *r*_clustermates (population) _is the relatedness among nestmates or clustermates with respect to allele frequencies in the population. *n *is the number of nests. Estimates significantly greater than zero are in bold.

The mitochondrial haplotypes showed strong and significant genetic differentiation among nests within clusters, and among clusters within populations (Table 1). The lowest genetic differentiation among clusters was also observed in the Chalet à Roch population (Table 1). Overall, the *F*-estimates for the mitochondrial haplotypes were four to six times greater than the ones for the nuclear makers, which indicates male-biased gene flow (see below; when both sexes disperse equally, a ratio *F*_st mitochondrial_/*F*_st nuclear _of approximately three is expected because of the smaller effective population size for the mitochondrial genome, [44]).

### Gene flow through males and females

The contrasting pattern of genetic differentiation between nuclear and mitochondrial markers revealed that the effective gene flow among nests was significantly male-biased in all three populations (Table 3). This result is conservative, as the gene diversity was slightly higher for mitochondrial markers than for nuclear ones (Table 3), which, other things being equal, should tend to decrease the *F*_st _for mitochondrial markers [46,47]. Rough estimates of relative gene flow through each sex suggest that the gene flow through males was on average three to nine times higher than the one through females, depending on the population (Table 3). The overall number of genetically effective migrants suggests that long-distance gene flow is restricted, given the very high number of queens per nest and presumably high level of genetic exchanges between neighbouring nests.

**Table 3:** Sex specific gene flow

Population	*F*_st nucl_	*F*_st mit_	*H*_s nucl_	*H*_s mit_	*N*_*m *_*m*_*m*_	*N*_*f *_*m*_*f*_	*N*_*m *_*m*_*m*_/*N*_*f *_*m*_*f*_
Bois de Peney	0.079 [0.051;0.097]	0.487	0.49	0.63	4.78 [3.6;8.3]	0.53	9.1 [6.8;15.7]
La Dôle	0.112 [0.081;0.149]	0.568	0.51	0.68	3.2 [2.1;4.9]	0.38	8.4 [5.5;12.9]
Chalet à Roch	0.049 [0.027;0.07]	0.202	0.54	0.60	5.76 [2.7;14.1]	1.97	2.9 [1.4;7.1]

Genetic differentiation among all nests within each population (*F*_st_) and gene diversity (*H*_s_) for the microsatellite and mitochondrial markers, as well as the corresponding estimates of male (*N*_*m *_*m*_*m*_) or female (*N*_*f *_*m*_*f*_) gene flow among nests. The ratio *N*_*m *_*m*_*m*_/*N*_*f *_*m*_*f *_provides a rough estimate of the relative contribution to gene flow of males, as compared to females. The 95% confidence intervals obtained by bootstrapping over microsatellite loci are indicated in brackets.

## Discussion

The evolution and maintenance of unicoloniality critically depends on patterns of dispersal and population genetic structuring, which in turn affect the degree of relatedness among nestmates. Here, we provide genetic evidence for cryptic genetic clustering in three unicolonial populations of the native wood ant *F. paralugubris*. This clustering is associated with male-biased gene flow, local exchanges between neighbouring nests and restricted long-distance gene flow.

The Bayesian analyses of the microsatellite genotypes revealed the presence of genetic clusters of nests within each of the three populations. Moreover, the use of a new model that incorporates geographic information permitted us to visualize the spatial location of cryptic genetic discontinuities within populations. In line with these findings, the *F*-statistics showed a Wahlund effect at the population level, as expected from the microgeographical genetic differentiation among nest clusters. In contrast, the genotypes of workers within clusters were in Hardy-Weinberg equilibrium, as expected from the model assumptions of the clustering methods. This analysis suggests that the clusters correspond to randomly mating breeding populations, with many queens and fairly high levels of local gene flow through queens and males, whereas gene flow between clusters is restricted.

The structure in clusters of nests has important consequences for the functioning and evolution of unicolonial populations. The crucial point is that clusters of nests are genetically differentiated, which results in moderate but significant levels of relatedness among members of the same cluster. In addition, nests within clusters are also slightly genetically differentiated, which further increases the relatedness among nestmates. The differentiation among nests within clusters may be explained by a finite number of queens per nest coupled with restricted queen and worker dispersal [22,42]. It is also possible that workers and queens tend to segregate along kin lines during colony budding [53].

The significant genetic structuring among nests within clusters and among clusters within populations in *F. paralugubris *contrasts with the absence of genetic differentiation within supercolonies of the extensively studied invasive and unicolonial argentine ant *L. humile *[15]. The absence of differentiation within native supercolonies of *L. humile *might be linked to their small size and extensive queen movement [9], whereas the very large supercolonies in the introduced range are probably out of equilibrium and affected by repeated human transport or habitat disturbance [15].

Beside *F. paralugubris*, several non-invasive ant species had significant genetic structuring within unicolonial populations [25,27-30,54]. The finding of genetic structuring within native unicolonial populations and the resulting elevated relatedness among nestmates despite high queen numbers per nest beg the question of the potential impact of kin selection on the maintenance of altruism in these systems.

The restricted effective gene flow and ensuing genetic differentiation among distant nests within populations may promote the maintenance of altruism in two ways. First, elevated relatedness due to population viscosity may contribute to the maintenance of altruistic sterile workers [34]. However, given the high number of queens and low relatedness among nestmates in our study populations, the long-term maintenance of altruism would require very large benefits to costs ratios, implying that the synergetic benefits of cooperation are huge (see equation 2.4 in [34]). Second, the maintenance of reproductive altruism could be explained by kin selection if competition occurs among distant, genetically differentiated nests, or among clusters [42,55,56]. Whether clusters actually compete for resources remains to be investigated. Competition among clusters does not seem to involve aggressive worker behaviour [33,49,57]. However, given the high number of queens and males that fly away from the nests in early summer, it seems likely that competition among clusters will occur after the dispersal flight, when males try to mate and queens seek to establish new colonies or to be adopted into nests [41,58,59].

The use of biparentally-inherited nuclear microsatellites and maternally-inherited mitochondrial markers allowed us to roughly estimate the amount of gene flow through males and females. Previous studies based on microsatellites had revealed limited long-distance gene flow and isolation by distance in the population at Chalet à Roch [22,42,60]. Our new analyses of three populations confirmed that long-distance gene flow is restricted. First, there was significant genetic structuring for both types of markers at all levels. Second, the number of genetically effective migrants among all nests within each population was moderate, given the high numbers of queens per nest and high rate of exchanges between neighbouring nests.

The comparatively much higher degree of differentiation at mitochondrial than nuclear markers further revealed that effective gene flow is male-biased in all three populations. These data indicate that queens are more philopatric and disperse less frequently or less far than males do. It is likely that male gene flow occurs mostly within clusters, which would explain both the Hardy-Weinberg equilibrium within clusters and significant genetic differentiation among clusters. Male-biased gene flow seems to be common in other polygnous ants in which queens and workers disperse by budding [18,44,61-63].

The presence of more than one mitochondrial haplotype in many nests suggests that queens are occasionally accepted into foreign nests. This result contrasts with kin selection models suggesting that colonies should only accept daughter queens [64]. However, it does not imply that gene flow through females is high, because, once established, matriline associations can persist indefinitely and spread in the population if queen number is high, queens are adopted into their natal nests and nest budding occurs, as is the case in our study species [22,42]. Multiple mitochondrial haplotypes per nest have been found in many ant species, which indicates that unrelated queens can associate in ants, be they unicolonial or not [44,65,66].

Overall, the data suggest a social organisation in large breeding units containing moderately related individuals. At the same time, the movement of workers among neighbouring nests and absence of aggression within populations [33,49] are clear signs of unicoloniality. The evolutionary pathway to this type of social organisation with no aggression, mixing of workers among neighbouring nests and restricted long-distance dispersal of queens and males remains unclear, and has been suggested to depend on both ecological and genetical factors [7,42,67,68].

The life-history of *F. paralugubris *is at least partly consistent with an 'ecological pathway' hypothesis by which unicoloniality is ultimately mediated by habitat saturation [7,42,67,69]. *F. paralugubris *has a sessile life-history in a stable habitat. Workers build large, long lasting nests. They also tend aphids, and these socially-expandable resources may in turn lead to increased nest densities. This progressive habitat saturation makes independent colony foundation increasingly difficult, promoting the reacceptance of queens by the maternal colony and establishment of new nests by budding, which in turn leads to the formation of large networks of nests [42]. In addition, the absence of aggression reduces the costs of territoriality, allowing higher worker densities and effective habitat monopolisation [16,70,71]. It has even been suggested that under certain ecological conditions, abandoning aggression while retaining some discrimination ability might be a first stage in the formation of supercolonies or unicolonial populations [7]. In line with this argument, the absence of aggression was not due to a complete lack of discrimination in *F. paralugubris *and *L. austriacus*, as shown by the significantly longer antennation bouts among non-nestmates [7,33].

## Conclusion

This study revealed restricted, male-biased gene flow and cryptic genetic clustering within three native unicolonial populations of wood ants. This peculiar form of social organisation might be driven by progressive habitat saturation that favours intranidal mating, acceptance of daughter queens and colony budding. The positive relatedness among clustermates suggests that kin selection may still contribute to the maintenance of altruism in unicolonial populations as long as competition occurs among clusters, which seems likely during dispersal flights. Whether kin selection will be strong enough to maintain altruistic worker behaviour over evolutionary times remains an open question.

## Methods

### Field collection

We focussed on three large populations of *F. paralugubris *in the Swiss Jura Mountains. We sampled workers from 20 nests at Bois de Peney, 20 nests at Chalet à Roch and 18 nests at La Dôle in July 2003. In addition, for the analysis of mitochondrial variability we sampled workers from two other populations in the Jura and two populations in the Swiss Alps (ten nests at Bois de Ban, seven nests at Champs Simon, nine nests at Château d'Oex, and two nests near Lac de l'Hongrin). All sites were situated between 950 and 1310 meters above sea level (see [33] for a map). We recorded the coordinates of each nest with a global positioning system (D-GPS; Garmin Ltd. Romsey, UK). The distances between nests ranged from 15 m to 76 km.

### Genetic analysis

We used the microsatellite genotypes of eight workers per nest that we had already obtained for a previous analysis of the large-scale population genetic structure of *F. paralugubris *[33]. The microsatellites include four loci developed for *F. paralugubris *(FL12, FL20, FL21, FL29, [72]) and seven loci originally developed for *Formica exsecta *(FE7, FE8, FE11, FE19, FE37, FE38, FE42, [73]). See [33] for details on microsatellite genotyping.

We screened for mitochondrial DNA variation by sequencing the cytochrome oxidase subunit I and II (COI-COII; 1368 bp) [GenBank: EU600788], the region of NADH dehydrogenase from subunit 4 to subunit 6 (ND4–ND6; 1497 bp with a gap of 100 bp) [GenBank: EU600793], and the region from ND6 to ND1, which includes the cytochrome *b *(2427 bp and 2430 bp) [GenBank: EU600789–EU600792]. The primers used to amplify the DNA are listed in Table 4. We designed primers for the COI-COII region on conserved regions in the alignment of sequences from *Apis mellifera*, *Formica fusca *and *F. truncorum*, except for the primer COI-RLR-*F *which came from Roehrdanz & Degrugillier [74]. We designed primers for the ND6 to ND1 and ND4 to ND6 regions on conserved regions in the alignment of sequences from *A. mellifera *and *F. exsecta*, except for the primers Cytb-Fe which came from Liautard & Keller [66].

**Table 4:** Primers to amplify the mitochondrial DNA of *F. paralugubris*

Locus	Forward primer	Reverse primer	Fragment size (bp)
COI-RLR	TTGATTTTTTGGTCATCCAGAAGT	TAGGTGAATTTGAATTTTGTAATG	~980
COI-II a	CGACGTTACTCCGAATACCC	TGGCCTTGAAGAAGAAATCG	~500
COI-II b	CAAAATTCAAATTCNCCNTATGA	CCNGGNGTTGAGTCTATTTT	~500

ND4–ND6 a	CAAATATGAAATAAATAAATTGG	GTTTGTGAAGGGGTTTTAGG	~760
ND4–ND6 b	AATTAAYAAAGTTAATCCTAAAACCCC	CGTATAGAGATAGATTTTATRGAACAG	~950

Cytb-Fe	CAGTTTAATTTCTAATGAACAAAC	GGATCTCTAAAAATATATGGG	~1030
ND6-ND1 a	ACATACCACAGGTTCATCAAATCC	CGAGGTTTATTACCTCGAATGCGTTATG	~810
ND6-ND1 b	AGTAACCCCAATCCATATTCAACC	AATAGGGTCTATGCGGTCAG	~1040
ND6-ND1 c	CATAACGCATTCGAGGTAATAAACCTCG	GTAGCATTTTTAACTTTATTAGAACG	~770

CbM3	TATTTTCCATTATTAAAATCTGTTC	AGTAAGAAATATTTACAGGTGAGGG	163/166
PB300 T/C	AAACTGACCGCATAGACCCTAT-T/C	GTGTTATGTTGATAAGGTGGG	171
PB30 A/G	CCCACCTTATCAACATAACAC	AATGCGGAAAGGTCCTAATAAGG-T/C	164

The first nine pairs of primers listed above were used for the initial screen of three mitochondrial regions (COI-COII, ND4-ND6 and ND6-ND1). The last three pairs of primers (CbM3, PB300 T/C and PB30 A/G) were used for the routine typing of three polymorphic sites in the ND6-ND1 region.

In the initial screen for polymorphism, we sequenced 11 workers sampled from 11 independent nests from the seven study populations. We extracted DNA from head and thorax with a standard phenol chloroform protocol. PCR products were purified using the QIAquick purification kit (Qiagen) and sequenced using the BigDye terminator ready-reaction kit and an ABI 3100 automated sequencer (Applied Biosystems). Sequence data were edited and compiled with the software Sequencher 3.0 (Gene Codes Corporation). To confirm the amplification of the target region of the mtDNA, the final sequences were aligned with the honeybee sequence [75] using the EMBL-EBI ClustalW v.1.83 website . We detected one deletion/insertion of three base pairs and two single nucleotide polymorphisms (SNPs) in the region between ND6 and ND1. We found no polymorphism in the other regions sequenced.

For the routine typing of the three polymorphic sites in the ND6 to ND1 region, we designed the specific primers CbM3, PB300 T/C and PB30 A/G (Table 4). The DNA of the workers was extracted with a Chelex protocol as described in Holzer *et al. *[33]. To analyze the three base pair deletion/insertion, we designed new primers labelled with fluorescent dyes (CbM3), which amplified a fragment that was either 163 or 166 bp long. The fragment was scored on an ABI 377XL sequencer. To genotype the two SNPs PB300 T/C and PB30 A/G, we used specific locked nucleic acids (LNA) primers (Sigma-Proligo). The PCR products were electrophoresed on a standard ethidium bromide 1.5% agarose gel. We routinely scored the mitochondrial haplotypes in the three largest populations (Bois de Peney, Chalet à Roch and La Dôle), using the same eight individuals as for the microsatellites. In the other populations, we genotyped two individuals per nest.

### Statistical analysis

To search for cryptic genetic structure among groups of nests within populations, we analysed the microsatellite data set with the methods implemented in the computer programs Geneland [52,76] and Structure v. 2 [77]. These individual-centred methods aim to detect cryptic genetic structure, admixed individuals and migrants on the basis of the fact that migrants will have multilocus genotypes that differ from the ones expected for native individuals [50].

Geneland divides up geo-referenced individuals into clusters corresponding to panmictic units without any *a priori *information on the shape and limits of these clusters [52,76]. The set of individuals are attributed to "virtual" clusters represented by random unions of convex polygons of a Voronoi tessellation. Allele frequencies are estimated iteratively and the spatial organization that best corresponds to panmictic units is identified with a Markov chain Monte Carlo procedure. Simulations showed that this method correctly identifies unconnected spatial groups that belong to the same panmictic cluster, as well as large and uniform clusters corresponding to homogenous populations [52]. We first determined the number of clusters (*k*) within the populations with a maximum number of clusters of ten. Each run was repeated ten times with 100000 iterations. To allow the assignment of individuals sharing the same nest to a different cluster, the 'uncertainty of coordinates' was set to 20, as neighbouring nests are often within a distance range of 20 m. Once *k *was determined, we re-run the program ten times with the fixed number of *k *inferred from the previous runs.

The computer program Structure detects the proportion of the genome of an individual originating from each inferred cluster without taking any spatial information into account [admixture analysis, [77]]. Individuals are assigned sequentially to clusters, or to two or more clusters if their genotypes indicate that they are admixed. We used the admixture model with a burn-in of 50000 iterations and 1000000 iterations to estimate the parameters. The procedure was repeated ten times for each *k*.

The relatedness among worker nestmates was estimated with the computer program Relatedness 5.0.8 [78]. Nests where weighted equally. Standard errors and 95% confidence intervals were obtained by jackknifing over nests for populations, and over loci for clusters. Relatedness was measured with respect to allele frequencies in clusters and in populations. This double way to measure relatedness is justified by the fact that part of the competition may be local and occur only within clusters, whereas some degree of competition may occur at the population scale. Specifically, the relatedness *r*_nestmates (population) _and *r*_clustermates (population) _estimate the probability by which an allele is more likely to be shared among nest- and clustermates than expected from the allele frequency in the population to which the nest or cluster belongs. The relatedness *r*_nestmates (cluster) _is the probability by which an allele is more likely to be shared among nestmates than expected from the allele frequency in the cluster.

The genetic differentiation at the microsatellite markers was further analyzed in a three-level hierarchical *F*-analysis of variance, as implemented in the computer program Hierfstat [79,80]. The three hierarchical levels were the individuals, nests, and clusters previously identified in the analysis of microsatellites with Geneland. We analysed each population separately. *F*_ind-nest _and *F*_ind-cluster _are the correlations of genes within individuals, as compared to random genes within the nest and cluster, respectively. *F*_ind-pop _is the correlation of genes within individuals, as compared to random genes within the population. *F*_nest-cluster _estimates the genetic differentiation among nests within clusters, and *F*_cluster-pop _the genetic differentiation among clusters within populations. The genetic differentiation at the mitochondrial DNA was investigated in a two-level hierarchical analysis, as implemented in the computer program Arlequin [81]. The two levels were the nests and clusters previously identified in the analysis of the microsatellites with Geneland.

The use of both nuclear and mitochondrial markers allowed us to roughly estimate the relative contribution of each sex to gene flow. We estimated the gene flow among nests within populations from the genetic differentiation among all nests within each population at the biparentally-inherited nuclear microsatellites (*F*_st nucl_) and maternally-inherited mitochondrial (*F*_st mit_) markers. Because more diverse genetic markers have lower *F*_st _(the maximal possible value of *F*_st _being given by 1-*H*_s_) [46,47], we compared the gene (or haplotype) diversity *H*_s _of microsatellite and mitochondrial markers. The relative strength of gene flow through females or males was calculated according to Seppä et al. [44], with the effective gene flow of females estimated as *N*_*f *_*m*_*f *_≈ (1 - *F*_st mit_)/(2 *F*_st mit_) [82], where *N*_*f *_is the number of females breeding in a population and *m*_*f *_the immigration rate. The gene flow of males was calculated as *N*_*m *_*m*_*m *_≈ [(1 - *F*_st nucl_)/(2 *F*_st nucl_)] - [(1 - *F*_st mit_)/*F*_st mit_)] by applying Wright's island model [83] modified for haplodiploid species [84]. Because our study system does not fulfil many of the assumptions of the island model, the estimates *N*_*f *_*m*_*f *_and *N*_*m *_*m*_*m *_should not be considered as actual number of migrants [48], but as relative estimates of gene flow through each sex.

## Authors' contributions

BH, LK and MC jointly conceived, designed and coordinated the study. BH carried out the sampling and laboratory analyses, analyzed the data and wrote a first draft of the manuscript. LK and MC supervised the analyses and MC finalized the manuscript. All authors read and approved the final manuscript.
